# Supplementary material for: Development and Characterization of Eudragit® EPO-Based Solid Dispersion of Rosuvastatin Calcium to Foresee the Impact on Solubility, Dissolution and Antihyperlipidemic Activity
Source: Pharmaceuticals (Basel). 2022 Apr 18;15(4):492. doi: 10.3390/ph15040492 (PMC9025505; doi:10.3390/ph15040492)
Supplement: Supplementary file 1 [file pharmaceuticals-15-00492-s001.zip › pharmaceuticals-1644250-supplementary.pdf]

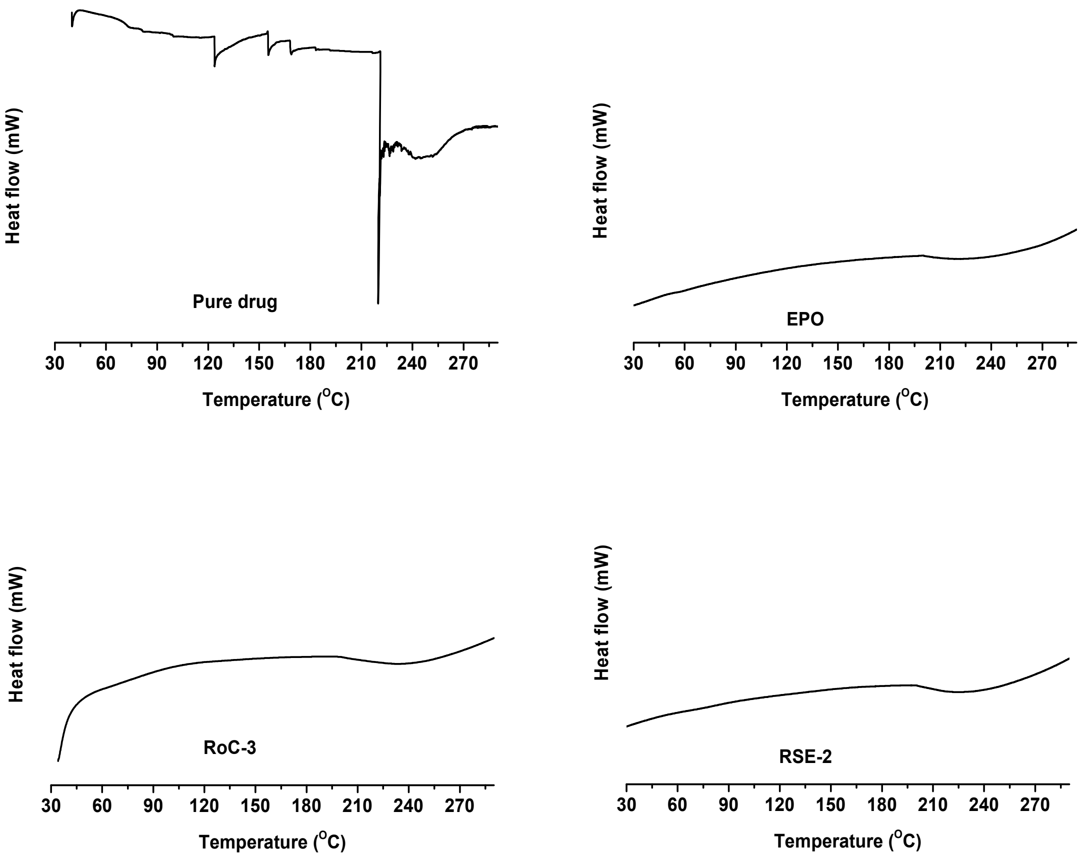

Figure S1. DSC of pure RoC, EPO and optimized formulations (RoC-3 and RSE-2).

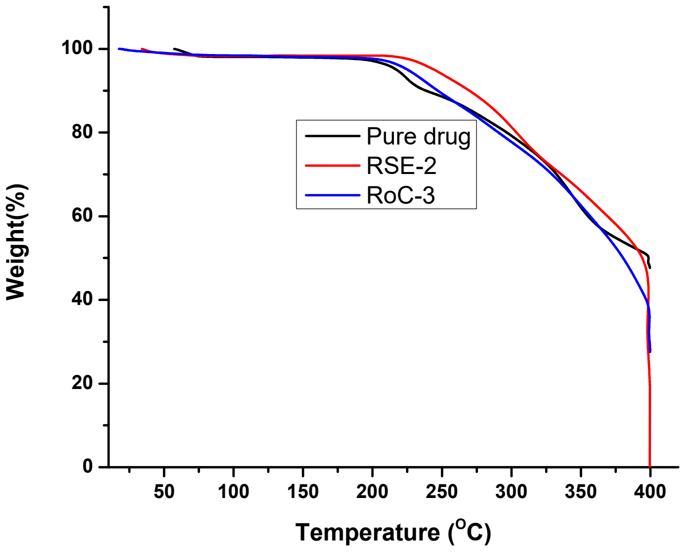

Figure S2. TGA thermogram of pure drug/RoC and optimized formulations (RoC-3 and RSE-2).

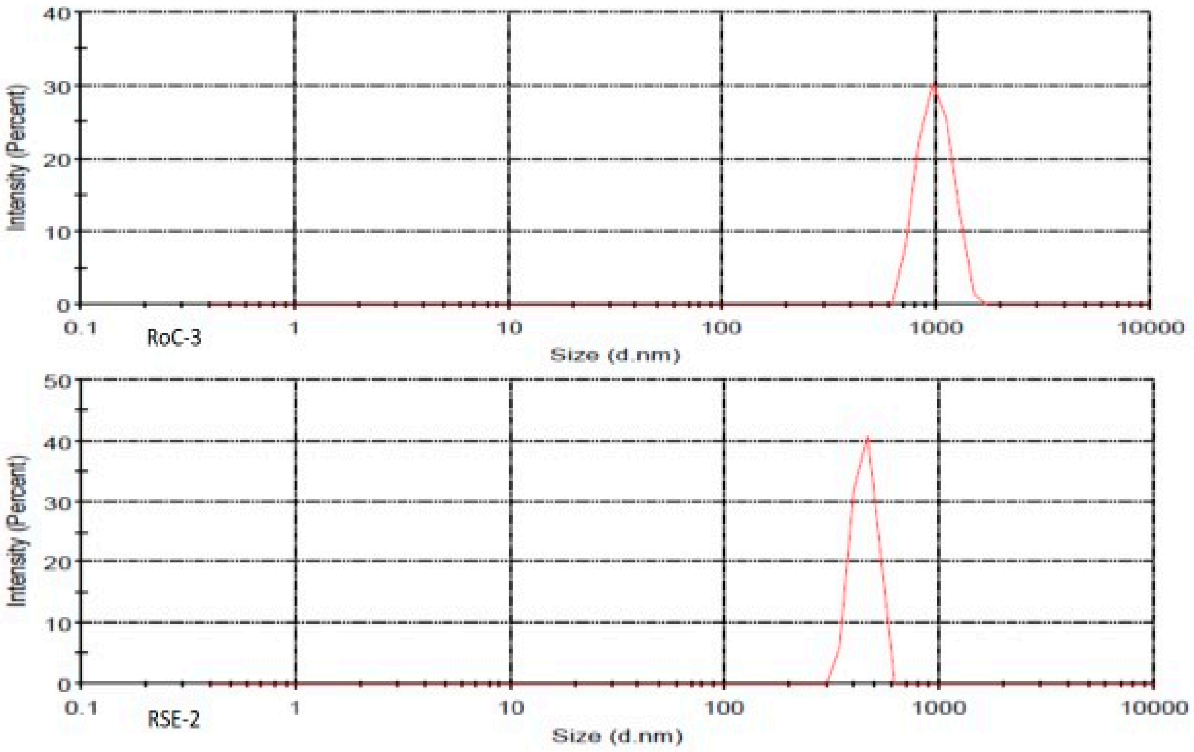

Figure S3. average particle size of RoC-3 and RSE-2.
